# Hepatoprotective activity of raspberry ketone against streptozotocin-induced type 2 diabetes in male rats

**DOI:** 10.1371/journal.pone.0324940

**Published:** 2025-06-09

**Authors:** Dalia Fouad, Doaa M. Elnagar, Ghaida Eid, Esraa Shuker

**Affiliations:** Department of Zoology, College of Science, King Saud University, Riyadh, Saudi Arabia; Jadavpur University, INDIA

## Abstract

Type 1 diabetes encompasses a spectrum of metabolic disorders marked by insulin deficiency, resulting in elevated blood glucose levels, commonly referred to as hyperglycemia. This persistent condition often precipitates lipid profile abnormalities, causing cholesterol alterations, low-and high-density lipoproteins, and triglycerides. The liver is particularly vulnerable to increased oxidative stress and inflammatory responses, which activate the transcription of pro-apoptotic genes and ultimately contribute to hepatocyte damage. This study analyzed the potential therapeutic role of raspberry ketone (RK), a natural antioxidant with antiapoptotic and anti-inflammatory properties, in male albino rats with induced type 2 diabetes. Fifty rats were equally divided into five groups: control, rats orally administered 200 mg /kg Body Weight (BW) RK for 5 days, diabetic rats intramuscularly injected once with 60 mg/kg BW streptozotocin, streptozotocin-induced diabetic rats orally administered 200 mg/kg BW RK for 5 days, and streptozotocin-induced diabetic rats orally administered 100 mg/kg metformin. Streptozotocin treatment significantly affected blood biochemical parameters, lipid profiles, oxidative stress markers, immunotoxicity biomarkers, and DNA damage biomarkers. Conversely, RK efficiently ameliorated the toxic effects of streptozotocin on the liver by reducing the pathological and biochemical changes associated with diabetes through its antioxidant and anti-inflammatory properties. Therefore, incorporating RK into the diet of diabetic patients can help prevent hepatocyte damage associated with diabetes. In conclusion, oral administration of RK exerts hepatoprotective effects by offering antioxidant, antiapoptotic, and anti-inflammatory properties against streptozotocin-induced type 1 diabetes in male rats.

## Introduction

Diabetes mellitus (DM) is a metabolic disorder characterized by inadequate insulin production or abnormal insulin response, resulting in high blood sugar (glucose) levels [1]. The International Diabetes Federation predicts that more than 300 million people will be affected by DM by 2025, highlighting the urgent need for improved treatment options for diabetes-related chronic disorders [2]. The majority of diabetes cases can be classified into two main categories: Type 1 diabetes (T1D) and type 2 diabetes (T2D). T1D is typically immune-associated, characterized by the destruction of insulin-producing pancreatic β cells. In contrast, T2D, which is more prevalent, is associated with insulin resistance and partial β-cell destruction, causing relative insulin deficiency [3]. T2D is among the most challenging epidemics due to its impact on human health and national economies [4]. The worldwide prevalence of T2D—which is largely caused by the complex interplay of genetic and environmental factors, including dietary and lifestyle factors—has more than doubled in the past 20 years [5]. Obesity-induced insulin resistance is known to accelerate pancreatic islet exhaustion, often heralding the onset of T2D [6,7].

Many complications of diabetes stem from chronic hyperglycemia and disruptions in lipid metabolism, including changes in cholesterol, low-density lipoproteins (LDL), high-density lipoproteins (HDL), and triglyceride levels. These abnormalities give rise to secondary complications, such as polyuria, polyphagia, polydipsia, ketosis, retinopathy, and cardiovascular diseases. These complications are primarily driven by increased production of reactive oxygen species (ROS) and diminished antioxidant defenses, leading to oxidative stress and tissue damage [8,9]. Effective control of plasma glucose levels is crucial in preventing diabetic complications and improving patients’ quality of life [10]. However, several hypoglycemic agents used in DM treatment have adverse side effects, including liver problems, lactic acidosis, and diarrhea [11]. Diabetes also leads to long-term damage, dysfunction, and failure of several organs, particularly the eyes, kidneys, nerves, heart, and blood vessels [12]. It alters the body’s cellular microenvironment, affecting important cellular processes such as apoptosis and inflammation. Furthermore, apoptosis affects pancreatic β cells, which are responsible for insulin production, contributing to the inability of the pancreas to compensate for insulin resistance in T2D [13]. Additionally, diabetes delays wound healing, a common side-effect that limits the repair of injured tissue [14]. Diabetes also activates cytokines, such as tumor necrosis factor (TNF-α), interleukin-1β, and interleukin-6, which promote inflammation [15]. Diabetic nephropathy, a microvascular complication, causes long-term or end-stage renal disease [16]. The pathophysiology of diabetes involves multifactorial interactions between lipid disorders, oxidative stress, renal hemodynamic changes, polyol activation, inflammatory pathways, and mitogen-activated protein kinase signaling pathways [17,18]. The ROS resulting from hyperglycemia leads to oxidative stress, damaging cells in various ways [19]. Oxidative stress is critical in the long-term complications of diabetes, accompanied by high peroxidation of lipids [20]. Augmented oxidative stress and changes in antioxidant capacity contribute to DM complications [17,21]. The clinical management of T2D requires an assessment of patients’ conditions and complications, incorporating lifestyle interventions, pharmacological therapy, and regular blood glucose monitoring [22]. With the increasing prevalence of diabetes, there is a pressing need for highly effective drugs with significantly reduced side effects. While current drugs reduce blood glucose levels, they often lead to obesity and hyperandrogenemia [23]. The major causes of oxidative stress in diabetes are disorders of the antioxidant enzyme system and inflammation, primarily caused by the excessive secretion of pro-inflammatory cytokines. These factors significantly contribute to the progression of DM and its complications. The prevention of these complications has been extensively researched, with medicinal herbs showing potential anti-inflammatory and antioxidant properties in disease prevention. Ketones from plants have been extensively studied for their pharmacological effects, including antidiabetic, antioxidant, and anti-inflammatory properties. Plant-based treatments are less toxic and cost-effective and have fewer side effects than synthetic medications—making them widely used for therapeutic purposes, including in DM management [12,24].

Raspberry ketone (RK) [4-(4-hydroxyphenyl)-2-butanone] is a natural phenolic compound responsible for the aroma and flavor of raspberries *(Rubus idaeus L.)*. RK has been used in various industries, such as perfumes and cosmetics, and as a flavoring agent in the food industry, for many years [25]. Recently, RK has gained attention for its hepatoprotective and antiapoptotic properties [26]. It also exhibits anti-inflammatory effects by blocking the NF-κB pathway and reduces abnormally high levels of malondialdehyde, a marker of oxidative stress [27]. RK has shown antiobesity effects by increasing fatty acid oxidation and lipolysis [28].

Raspberries have been used in food and medicine for centuries. They are rich in essential vitamins, minerals, and nonessential compounds, making them a valuable source of dietary antioxidants [29]. Raspberry consumption has been associated with reduced inflammation and cardiovascular disease risk factors, as well as protective actions against certain malignancies [30]. The ingestion of raspberry food products can lower body weight gain obesity associated with obesigenic high-calorie, high fat diet [31].

The present study investigates the potential effects of RK on streptozotocin-induced diabetic male albino rats as an antioxidant, anti-inflammatory, and antiapoptotic agent against diabetes.

## Materials and methods

### Experimental animals and chemicals

In this study, 50 healthy male albino rats weighing 188 ± 5 g (about 3 months old were obtained from the Animal House of King Saud University, Riyadh, Saudi Arabia, and housed under standard conditions (temperature, humidity, and a 12/12-h light/dark cycle) with free access to commercial pellet diet and tap water. They were acclimatized for 1 week before the initiation of the experiment. All animals were handled and sacrificed by the recommendations of the Ethics Committee of the university (KSU-SE-21–53).

Both streptozotocin 1 gm (chemical formula: C_8_H_15_N_3_O_7_) and RK [4-(4-hydroxyphenyl)-2-butanone 99%] were obtained from Sigma-Aldrich. Commercial assay kits were obtained from Cayman Chemical Company (AnnArbor, MI, USA) and Thermo Scientific (USA) and used to measure the antioxidant defense enzyme, oxidative stress markers, and lactate dehydrogenase (LDH). The DNA extraction kit was procured from Qiagen (Hilden, Germany). Other chemicals used were high-grade analytical reagents.

### Induction of diabetes

Streptozotocin was dissolved in freshly prepared 0.05 M citrate buffer (pH 4.5). The animals were subjected to fasting overnight for 12 h before being injected with a single dose of streptozotocin (60 mg/kg BW) intraperitoneal. Diabetes was confirmed after three days by measuring fasting blood glucose levels from the rats’ tail veins by using a glucometer (Accu-Check Performa, Mannheim, Germany) [32,33].

### Experimental design

The animals were equally divided into five groups (n = 10) based on their treatments:

Group 1: Control group: treated orally with saline.
Group 2: Treated orally with RK (200 mg/kg BW) for 5 days [26].
Group 3: Injected with a single intraperitoneal dose of streptozotocin (60 mg/kg BW), as in the induction of diabetes.
Group 4: Injected with a single intraperitoneal dose of streptozotocin (60 mg/kg BW) and orally treated with only RK (200 mg/kg BW) for 5 days after diabetes was confirmed.
Group 5: Injected with a single intraperitoneal dose of streptozotocin (60 mg/kg BW) and orally treated with metformin (100 mg/kg BW) for 5 days after diabetes was confirmed [33].

After 5 days of treatment, five rats from each group (control and treated) were sacrificed at 1-h and 24-h intervals. The rats were sacrificed using intraperitoneal injections of pentobarbital (250 mg/kg), according to the previous study [32]. The rats were killed by intraperitoneal injection of pentobarbital to prevent further suffering. After that, the skin of the animals was incised along the ventral midline from the sternum to the pubis and reflected using blunt dissection. The linea alba was incised, and the muscles along the costal arch were cut to expose the peritoneal cavity.

### Sample preparation

Immediately after decapitation, blood was collected from the rats’ trunks in a serum separator tube for biochemical analysis. The blood samples were collected in a sterile, closed plain tube and allowed to clot at 25°C. Then, the tubes were centrifuged at 3500 rpm and 4°C for 15 min. The serum samples were transferred to sterile Eppendorf tubes and stored at −80°C until further analysis. For histological and immunohistochemistry studies, the liver was immediately removed and perfused with ice-cold saline. Small liver pieces were transferred to suitable fixatives (10% buffered formaldehyde). For analyzing antioxidant defense enzymes and oxidative stress markers, a part of the liver was weighed and homogenized at a 1:10 (w:v) ratio in phosphate buffer saline (pH 7.4). For DNA extraction, a part of the liver was immediately stored at −80°C.

### Measurement of blood glucose level

The blood samples were taken daily from the tail vein of the animals under anesthesia. The Accutrend Plus System and Accutrend blood strips were used to measure blood glucose levels [33].

### Lipid profile

The total cholesterol, LDL cholesterol, HDL cholesterol, and triglyceride levels and activities in the rats’ serum were evaluated using assay kits (Sigma Chemical Co, St Louis, MO, USA) [34].

### Liver function test

The alanine aminotransferase (ALT) and aspartate aminotransferase (AST) levels and activities in the rats’ serum were determined using a Reflotron Plus dry chemistry analyzer (Roche, Germany).

### Histopathological examination

The liver samples from the control and treated groups were fixed in 10% buffered formaldehyde for 24 h and then washed with tap water. Subsequently, they underwent standard procedures for dehydration, clearing, and paraffin embedding. The liver was cut into 5-μm-thick sections using a rotary microtome and stained with hematoxylin and eosin [35].

### Antioxidant defense enzyme and oxidative stress biomarkers

The liver was weighed and homogenized in a medium according to the protocol. Lipid peroxidation and nitric oxide (NOx) were determined using commercial kits (Cat. No. B3932, EMSNO, Thermo Fisher Scientific, USA), according to a previously described method [36]. Reduced glutathione was determined in serum and estimated using Elman’s reagent (5,5-dithiobis-2-nitrobenzonic acid) following the method described in [37], with some modifications. Superoxide dismutase (SOD) and catalase were measured as antioxidant enzymes using Cayman Chemical Company’s commercial assay kits (Cat. No. 706002 and 707002, Ann Arbor, MI, USA).

### Determination of LDH

Extracellular lactate dehydrogenase, an index of necrosis, was measured using commercial assay kits (Cat. No. 601170, Cayman Chemical Company, Ann Arbor, MI, USA).

### Total DNA preparation, extraction, and fragmentation

DNA was extracted from the liver tissue using the DNA Mini Kit (CAT. No. 80204, Qiagen) according to the manufacturer’s instructions. The quantitation of DNA was evaluated using a Nanodrop spectrophotometer. Agarose gel electrophoresis was performed on a 1%–2% agarose gel, which was then illuminated with 300-nm UV light and photographed [38].

### Immunohistochemistry

Formalin-fixed tissues were embedded in paraffin and sectioned into 5-μm-thick slices. These liver sections were left to mount overnight, then deparaffinized in xylene and rehydrated through a series of decreasing ethanol concentrations. Subsequently, they were subjected to antigen unmasking by boiling for 5 minutes. The slides were immersed in a peroxidase-blocking reagent for 10 min, followed by 30-min incubation in a humidified chamber with blocking goat serum (Dako). Immunohistochemistry, using monoclonal as well as polyclonal antibodies, was employed to analyze the tissue distribution of the antigens of interest (P53) in both healthy and diseased tissues [39].

### Statistical analysis

Data were analyzed using SPSS (ver.29; SPSS Inc., Chicago, IL, USA). All values were expressed as the mean ± standard error. A *p*-value of <0.05 was considered significant.

## Results

### Body weight and blood glucose

**Fig 1:**
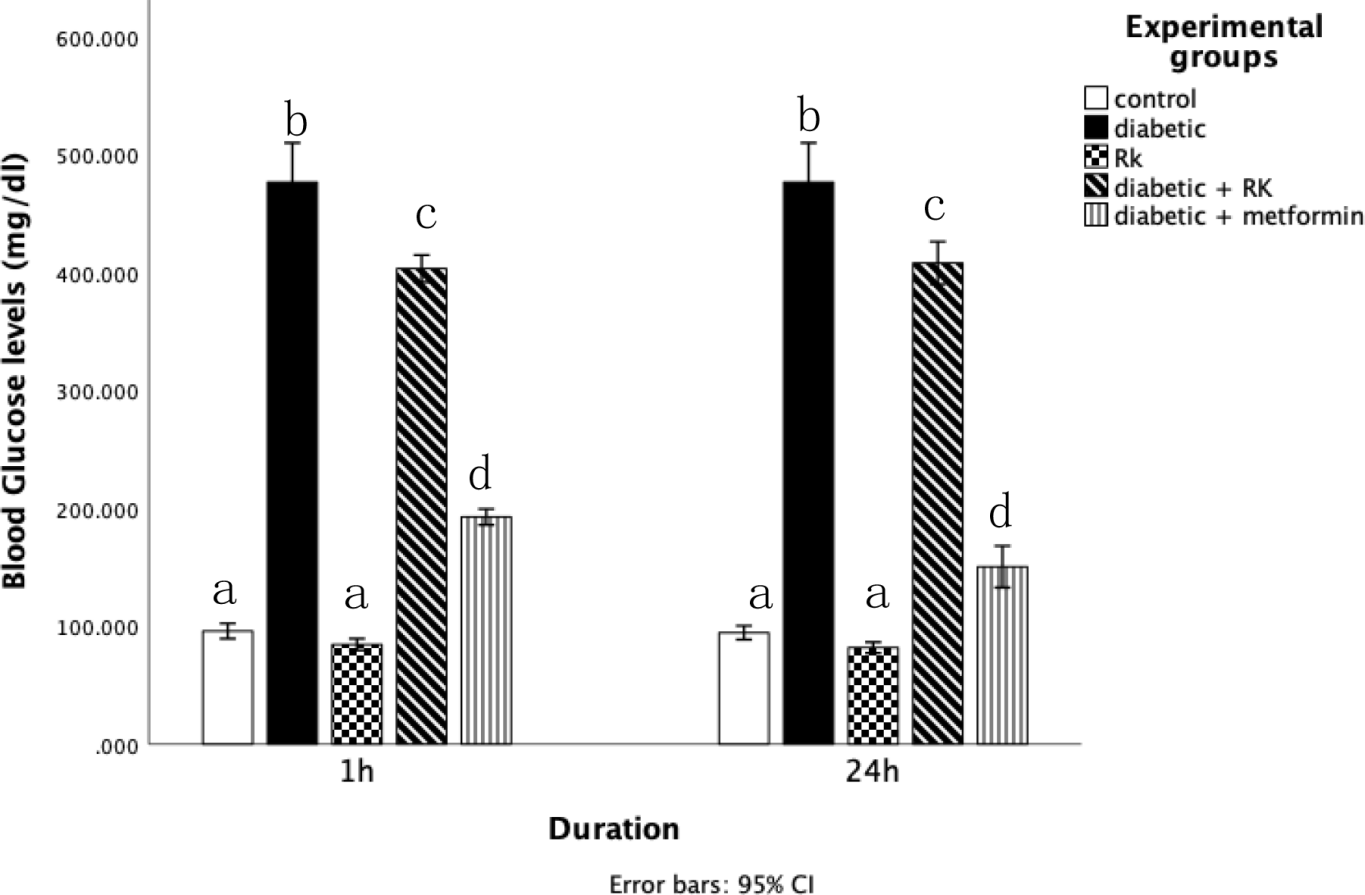
Effects of raspberry ketone and metformin- on blood glucose (mmol/L) levels on the day of dissection in control and experimental rats. Similar superscripts indicate non-significant difference; different superscripts indicate significant difference.

Throughout the experimental period, no significant changes were noted in the body weight of diabetic rats treated with RK or metformin for 1 week. To confirm the diabetic model and the treatment (RK or metformin) effect, the animals’ blood glucose levels were checked on the day they were dissected (Fig 1). The blood glucose level of the diabetic rats was significantly higher than that of the controls. However, after treatment with RK or metformin for 10 days, their blood glucose level decreased significantly.

### Lipid profile

No significant difference was noted in serum cholesterol levels among all groups. However, serum triglyceride, HDL, and LDL levels were significantly higher in the diabetic group than in the control group and the group treated with only RK (p ≤ 0.05). These levels were significantly reduced in the diabetic groups treated with RK or metformin compared to the diabetic group (Table 1), which indicated its hepatoprotective effects.

**Table 1:** Effects of raspberry ketone-and metformin on serum cholesterol, triglycerides, high-density lipoproteins, and low-density lipoproteins levels (U/L) in control and diabetic rats.

Group parameters	Control rats	Diabetic rats	RK	Diabetic + RK	Diabetic + metformin
**LDL (mg/dL 1 h)**	65.6 ± 2.12^a^	80.62 ± 1.58^b^	65.7 ± 0.75^a^	75.76 ± 1.39^c^	69.42 ± 1.17^a^
**LDL (mg/dL 24 h)**	64.52 ± 1.25^a^	79.5 ± 1.8^b^	68.35 ± 1.49^a^	73.65 ± 1.06^c^	69.79 ± 1.82^a^
**HDL (mg/dL 1 h)**	25 ± 0^a^	35.34 ± 0.64^b^	26.68 ± 0.89^a^	28.1 ± 1.73^c^	29.66 ± 1.37^d^
**HDL (mg/dL 24 h)**	25.6 ± 0.4^a^	36 ± 1.28^b^	26.8 ± 1.2^a^	31.15 ± 1.45^c^	33.60 ± 0.46^d^
**Cholesterol (mg/dL 1 h)**	91.4 ± 0.74^a^	97 ± 0.70^b^	93.4 ± 1.36^a^	96 ± 1.04^c^	94 ± 1.22^a^
**Cholesterol (mg/dL 24 h)**	92 ± 0.7^a^	98.2 ± 0.37^b^	93.4 ± 0.92^a^	94.4 ± 0.74^c^	95.8 ± 0.86^a^
**Triglycerides (mg/dL 1 h)**	64 ± 1.30^a^	83.74 ± 1.88^b^	64.72 ± 1.37^a^	80.12 ± 2.30^c^	76.80 ± 0.68^c^
**Triglycerides (mg/dL 24 h)**	64.3 ± 0.91^a^	84.66 ± 1.78^b^	63.28 ± 1.48^a^	78.28 ± 0.70^c^	75.96 ± 0.98^c^

HDL, high-density lipoproteins; LDL, low-density lipoproteins; RK, raspberry ketone; Similar superscripts indicate non-significant difference; different superscripts indicate significant difference.

### Liver function

**Fig 2:**
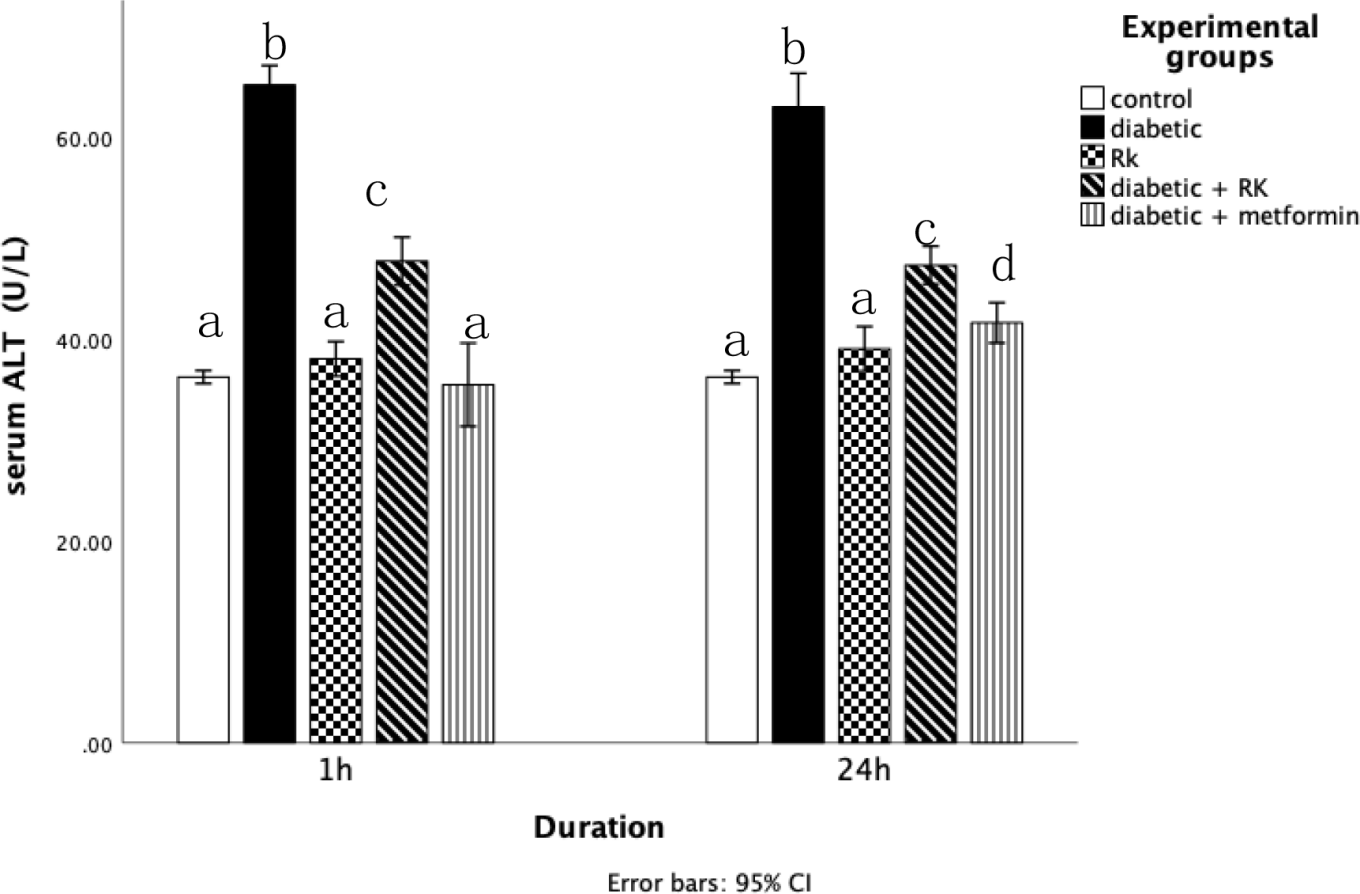
Effects of raspberry ketone and metformin on alanine aminotransferase (U/L) levels in control and experimental rats. Similar superscripts indicate non-significant difference; different superscripts indicate significant difference.

**Fig 3:**
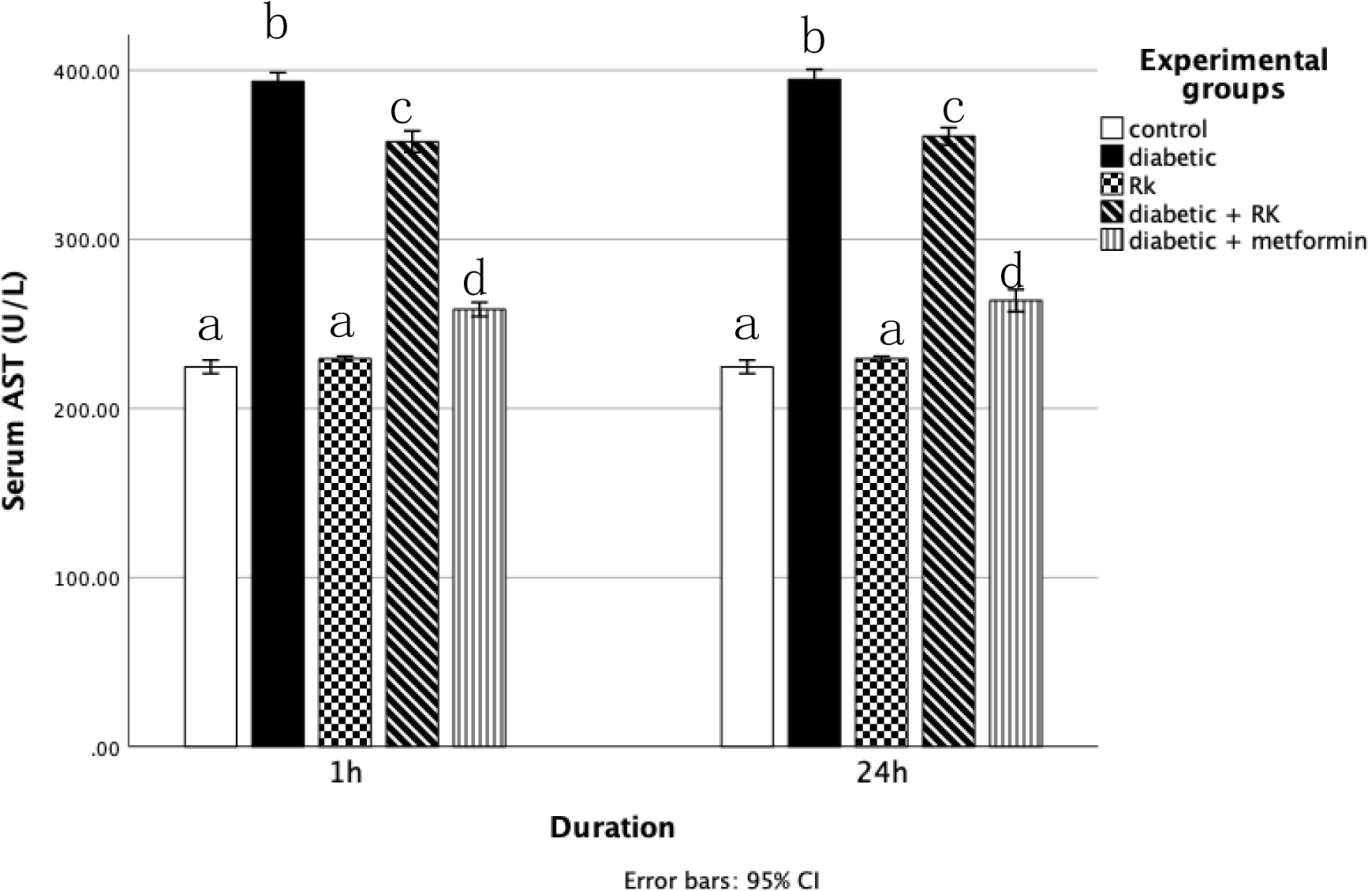
Effects of Raspberry ketones and metformin on aspartate aminotransferase (U/L) levels in control and experimental rats. Similar superscripts indicate non-significant difference; different superscripts indicate significant difference.

The ALT and AST activity levels were significantly higher in the diabetic group than in the control group and the group treated with only RK. However, they were significantly decreased in the groups treated with RK or metformin compared to the diabetic group (p ≤ 0.05) (Figs 2 and 3), which proved that RK is hepatoprotective.

### Oxidative stress markers

**Fig 4:**
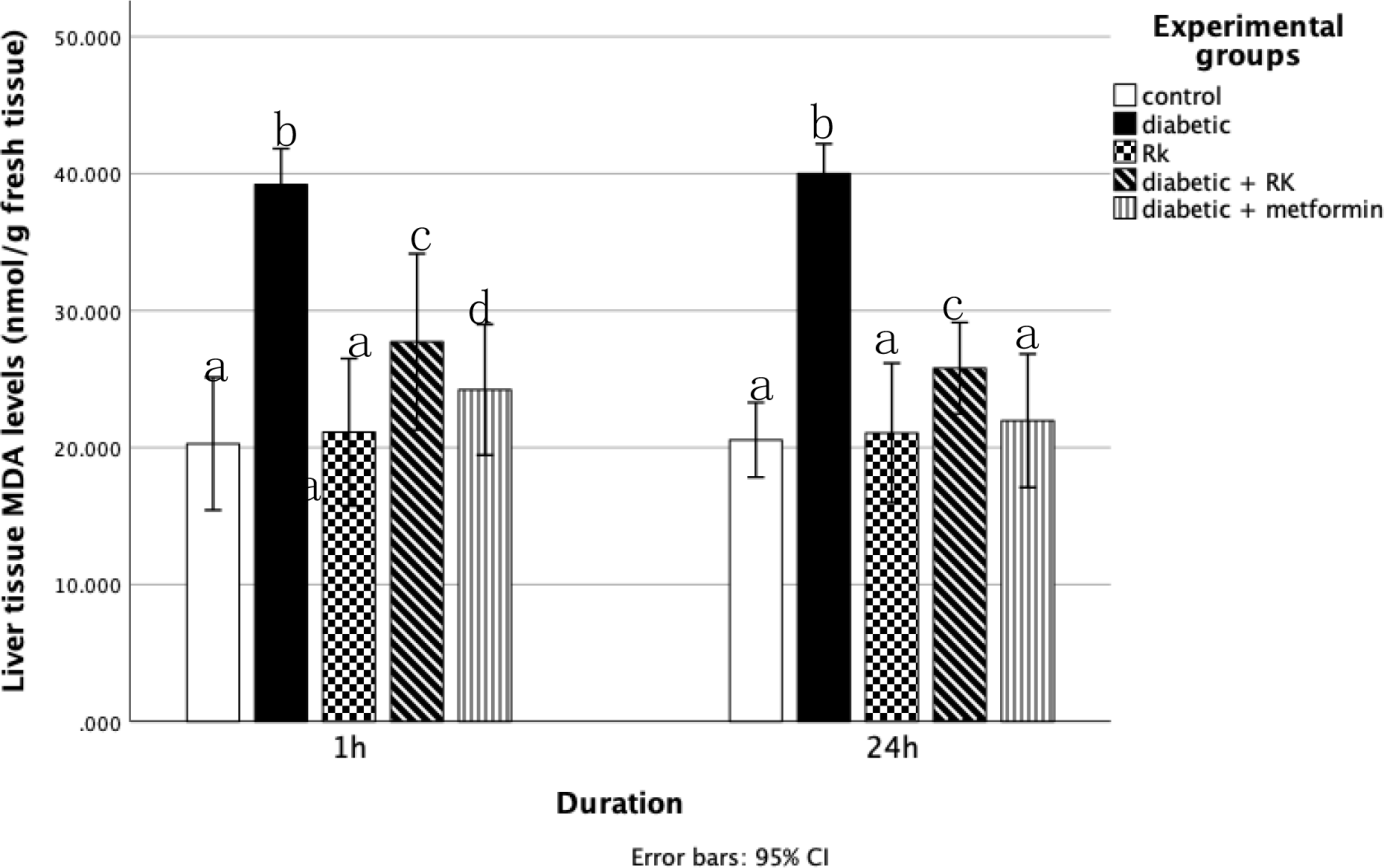
Effects of raspberry ketone and metformin on malondialdehyde (U/g) levels in the livers of control and experimental rats. Similar superscripts indicate non-significant difference; different superscripts indicate significant difference.

**Fig 5:**
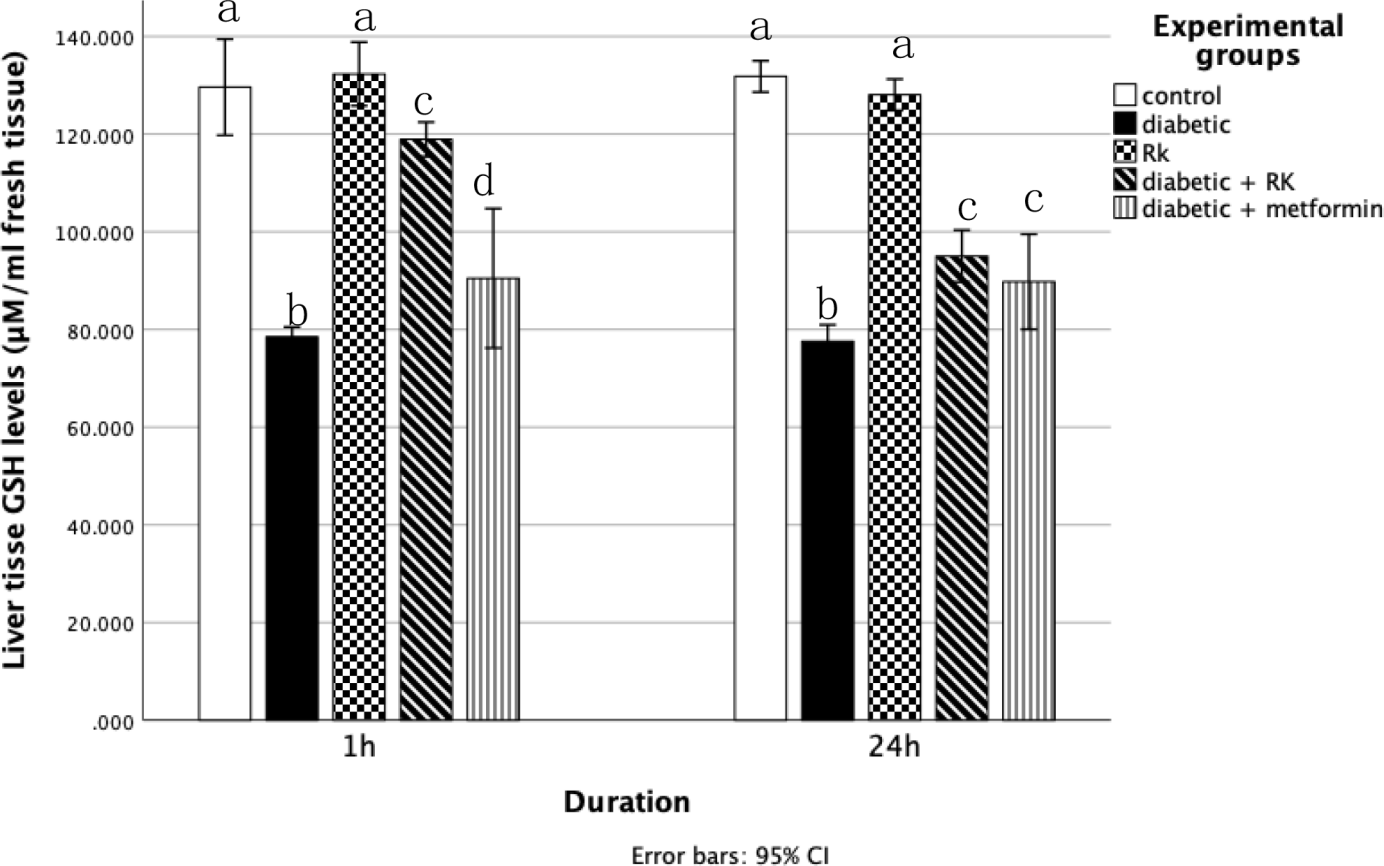
Effects of raspberry ketone and metformin on reduced glutathione (nmol/g) levels in the livers of control and experimental rats. Similar superscripts indicate non-significant difference; different superscripts indicate significant difference.

Compared to all other groups, the diabetic group had significantly higher serum malondialdehyde and NOx levels and significantly decreased glutathione levels in the liver tissue homogenates. Rats treated with only RK showed significantly decreased malondialdehyde and NOx levels in their liver compared to the control group. The group treated with RK or metformin for 1 h and 24 h had significantly reduced malondialdehyde and NOx levels in their liver compared to the diabetic group (Fig 4 and Table 2). However, the group treated with only RK for 1 h had significantly increased glutathione activity compared to the group treated with only metformin. The group treated with only RK for 24 h had slightly increased glutathione activity compared to the group treated with metformin for 24 h (Fig 5).

**Table 2:** Effects of raspberry ketone-and metformin on nitric oxide (µmol/L) levels in control and experimental rats.

Group parameters	Control	Diabetic	RK	Diabetic + RK	Diabetic + metformin
**Nitric oxide (nmol/g) 1 h**	1.31 ± 0.04^a^	2.49 ± 0.08^b^	1.02 ± 0.03^c^	1.69 ± 0.09^d^	1.40 ± 0.08^a^
**Nitric oxide (nmol/g) 24 h**	1.37 ± 0.16^a^	2.45 ± 0.06^b^	0.92820 ± 0.03^c^	1.59 ± 0.1^d^	1.47 ± 0.05^a^

RK, raspberry ketone; Similar superscripts indicate non-significant difference; different superscripts indicate significant difference.

### Antioxidant enzymes

**Fig 6:**
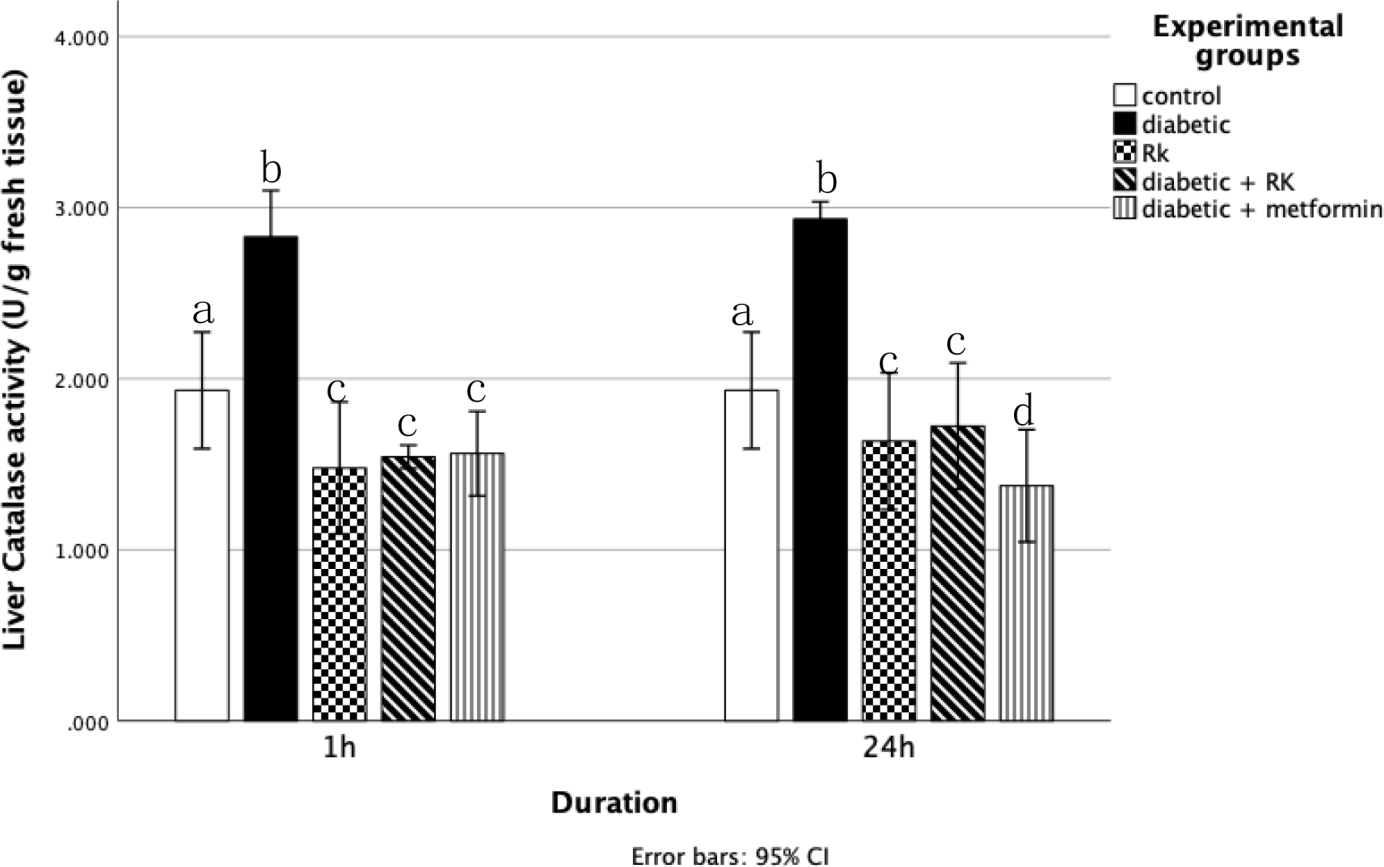
Effects of raspberry ketone or metformin on catalase (U/g) levels in the livers of control and experimental rats. Similar superscripts indicate non-significant difference; different superscripts indicate significant difference.

**Fig 7:**
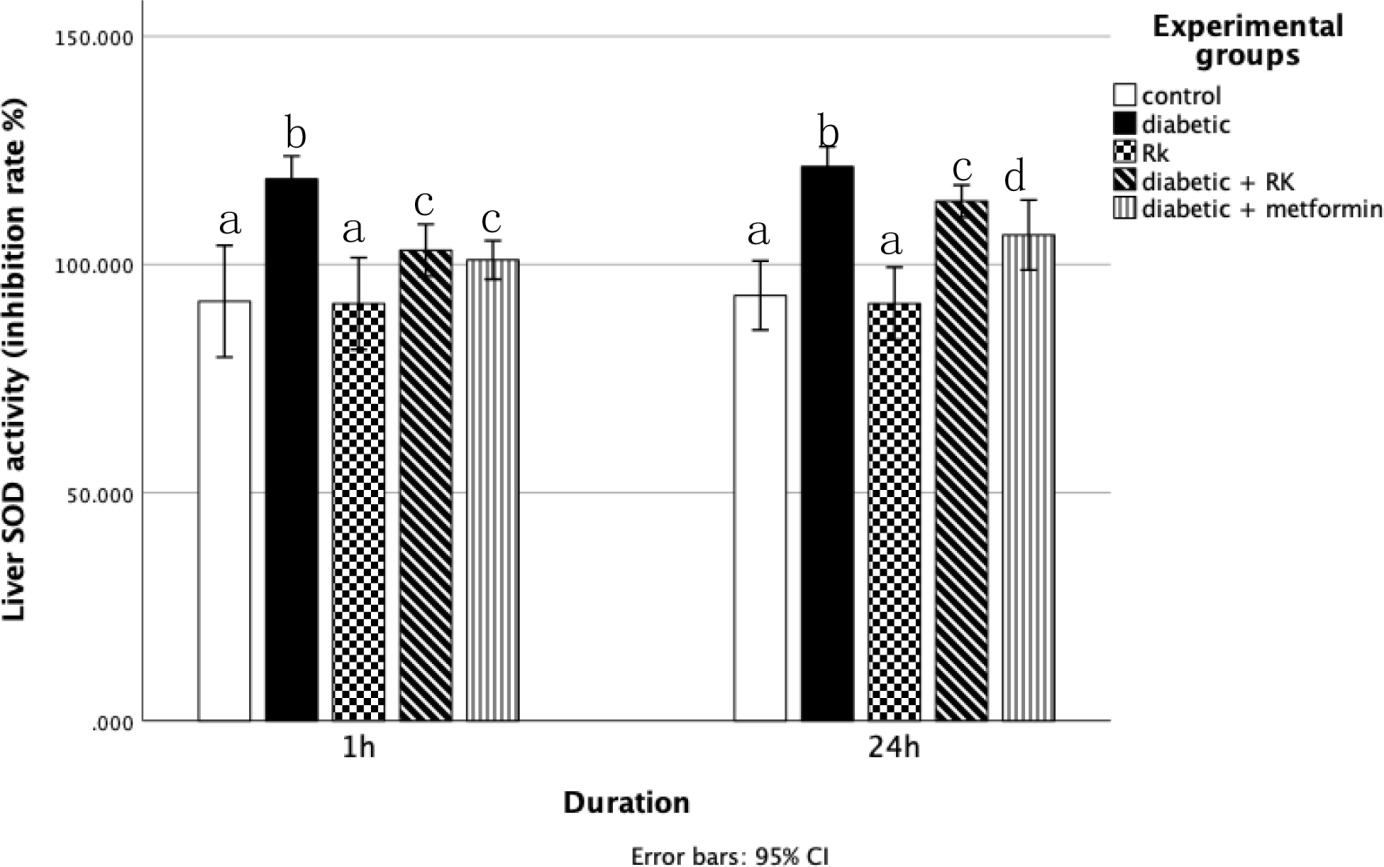
Effects of raspberry ketone and metformin on superoxide dismutase (U/mL) activity levels in the livers of control and experimental rats. Similar superscripts indicate non-significant difference; different superscripts indicate significant difference.

The catalase and SOD activity levels in the liver tissue homogenates were significantly higher in the diabetic group compared to the control group. Rats treated with only RK showed a significant (p ≤ 0.05) decrease in catalase activity compared to the control group (Fig 6). Conversely, rats treated with RK or metformin for 1 h and 24 h had significantly decreased catalase and SOD levels compared to the diabetic group. The group treated for 1 h had more decreased catalase and SOD levels than the group treated for 24 h (Fig 7).

### LDH levels

The serum LDH levels were significantly higher in the diabetic group than in the control group. The groups treated with RK or metformin for 1 h and 24 h had significantly decreased LDH levels compared to the diabetic group (Table 3).

**Table 3:** Effects of raspberry ketone-and metformin on lactate dehydrogenase (µmol/L) levels in control and experimental rats.

Group parameters	Control	Diabetic	RK	Diabetic + RK	Diabetic + metformin
**Lactate dehydrogenase (UI/L) 1 h**	1.95 ± 0.07^a^	3.48 ± 0.08^b^	1.85 ± 0.03^c^	2.77 ± 0.04^d^	2.28 ± 0.08^e^
**Lactate dehydrogenase (UI/L) 24 h**	2.02 ± 0.07 ^a^	3.20 ± 0.07^b^	1.80 ± 0.05^c^	2.73 ± 0.03^d^	2.06 ± 0.06^a^

RK, raspberry ketone; Similar superscripts indicate non-significant difference; different superscripts indicate significant difference.

### DNA fragmentation

**Fig 8:**
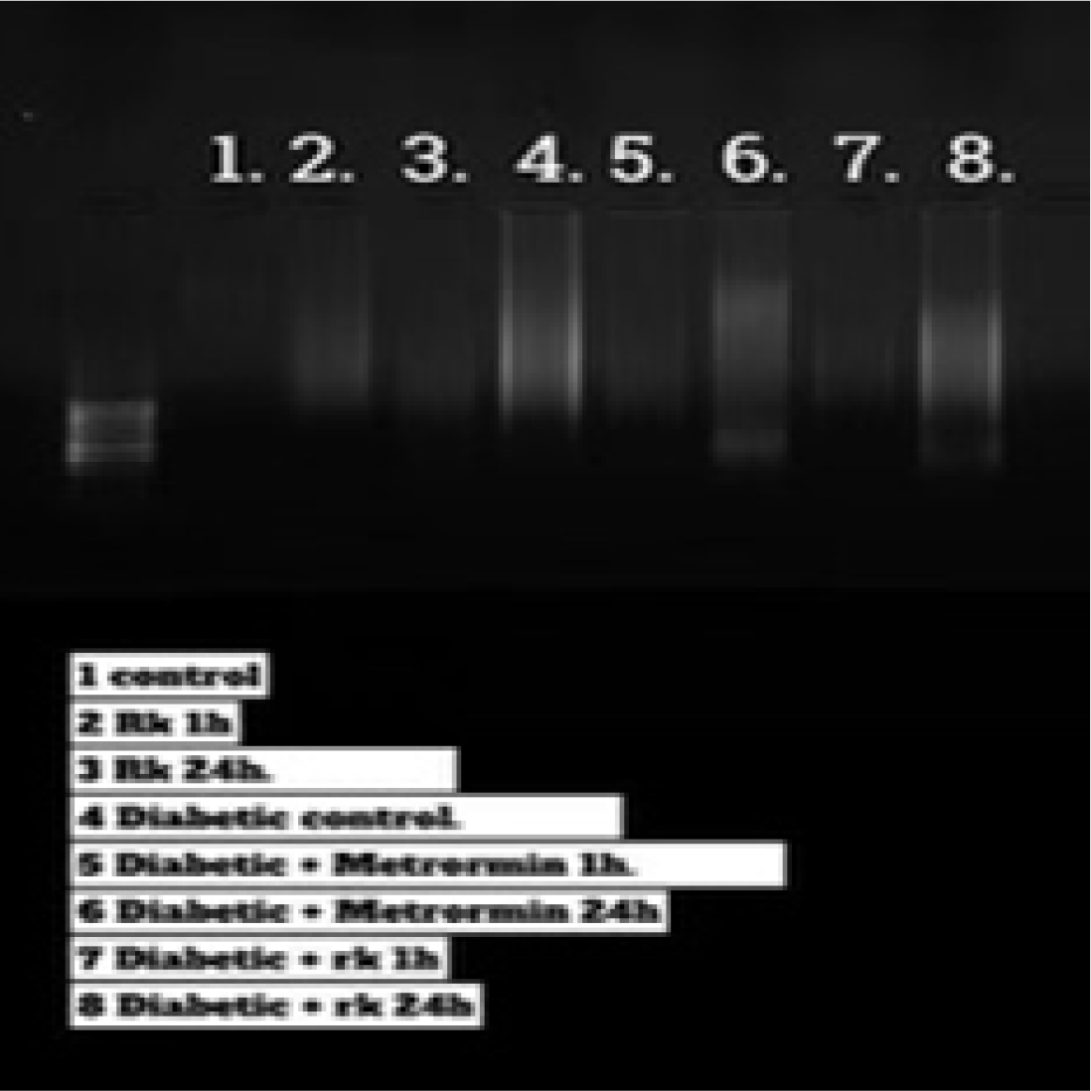
DNA fragmentation in control and experimental rats. Lane 1: control group; lane 2: rats treated with only raspberry ketone (RK) for 1 h; lane 3: rats treated with only raspberry ketone (RK) for 24 h; lane 4: diabetic group (50 mg/kg); lane 5: diabetic rats treated with metformin (50 mg/kg) for 1 h; lane 6: diabetic rats treated with metformin (50 mg/kg) for 24 h; lane 7: diabetic group treated with RK (200 mg/kg BW) for 1 h; lane 8: diabetic rats treated with RK (200 mg/kg BW) for 24 h.

DNA fragmentation, a marker of apoptosis, was assessed qualitatively by examining the integrity of genomic DNA extracted from the liver tissues of male rats (Fig 8). Agarose gel electrophoresis revealed that the DNA extracted from the control rats (lane 1) and rats treated with 200 mg/kg RK (lanes 2 & 3) showed high-quality DNA compared to the diabetic group (lane 4). However, the diabetic group treated with RK or metformin showed less DNA damage.

### Histopathological examination

**Fig 9:**
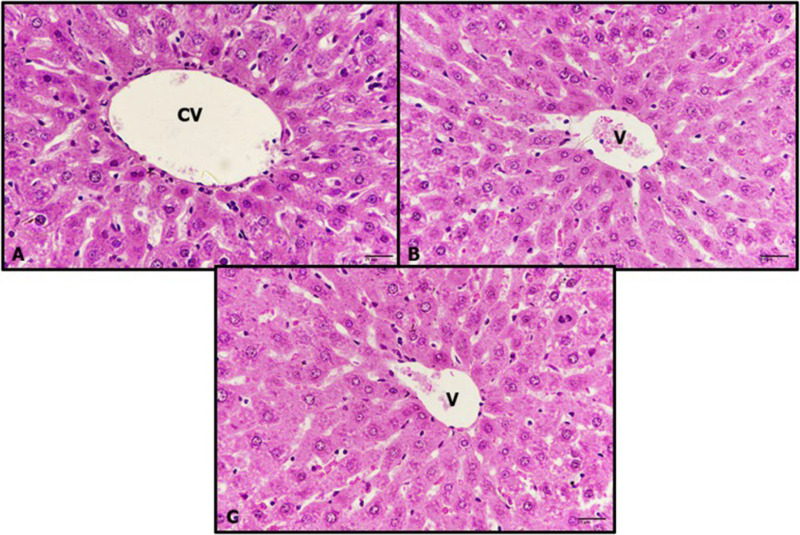
Photomicrographs of rats’ livers. (A) untreated control rats, (B) livers of rats treated with only raspberry ketone (RK) for 1 h, (C) livers of rats treated with only RK for 24 h (hematoxylin and eosin, 400×).

**Fig 10:**
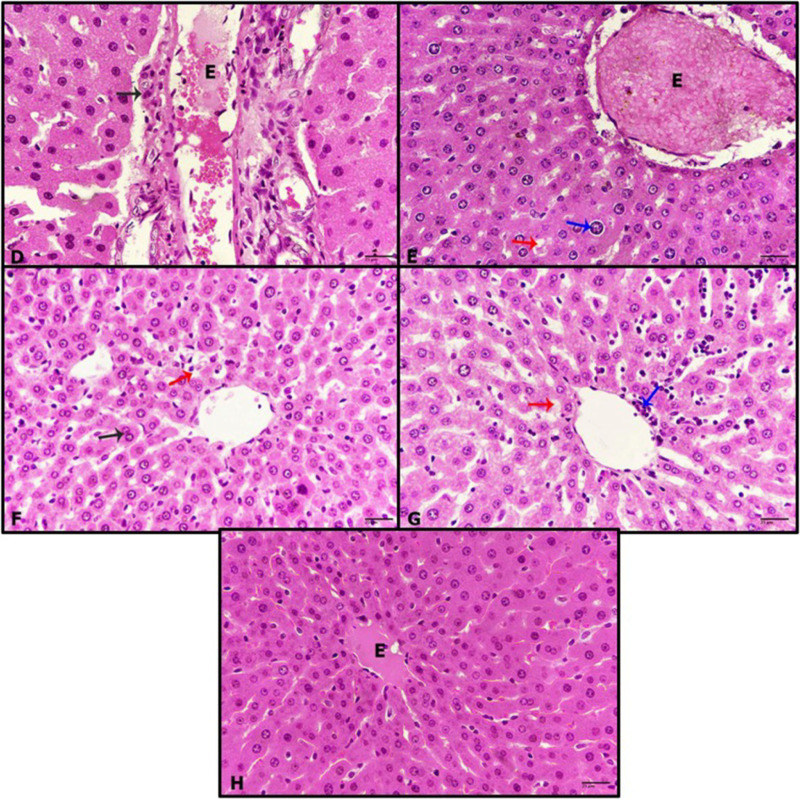
Photomicrographs of rat livers. (D) diabetic rats showing dilated vein congested with edema (E) and surrounded with inflammatory cells (black arrow), (E) diabetic rats treated with raspberry ketone (RK) for 1 h revealing congested vein with edema (E), cytoplasmic degeneration (red arrow), swelling nucleus (blue arrow), (F) diabetic rats treated with RK for 24 h displaying cytoplasmic degeneration (red arrow), binucleated cell (black arrow), (G) diabetic rats treated with metformin for 1 h revealing cytoplasmic degeneration (red arrow), infiltrative cells (blue arrow), (H) diabetic rats treated with metformin for 24 h showing congested vein with edema (E), healthy hepatocytes (hematoxylin and eosin, 400×).

The livers of untreated control rats exhibited a normal structure of hepatic tissue, with central veins and strands of hepatocytes separated from each other by blood sinusoids (Fig 9A). After the diabetic rats were treated with RK for 1 and 24 h, their livers showed a normal structure resembling that of the controls (Figs 9B and C). However, the livers of the diabetic rats displayed significant pathological signs, including dilatation of veins congested with edema and hemorrhage, as well as inflammation (Fig 10D). Additionally, diabetic rats treated with only RK for 1 h showed limited improvement, with alterations such as vein congestion with edema, cytoplasmic degeneration, and enlargement of hepatocyte nuclei (Fig 10E). Diabetic rats treated with only RK for 24 h showed more improved hepatic tissues, with binucleated cells, indicating regeneration (Fig 10F). However, after the diabetic rats were treated with metformin for 1 h, they exhibited pathological features, such as cytoplasmic degeneration, sinusoid dilatation, and inflammation (Fig 10G). Furthermore, after the rats were treated with metformin for 24 h, significant improvement was observed in their hepatic cells, although their veins remained congested with edema (Fig 10H). These results showed the hepatoprotective effects of RK against streptozotocin-induced type 2 diabetes in male rats.

### Immunohistochemistry (p53)

**Fig 11:**
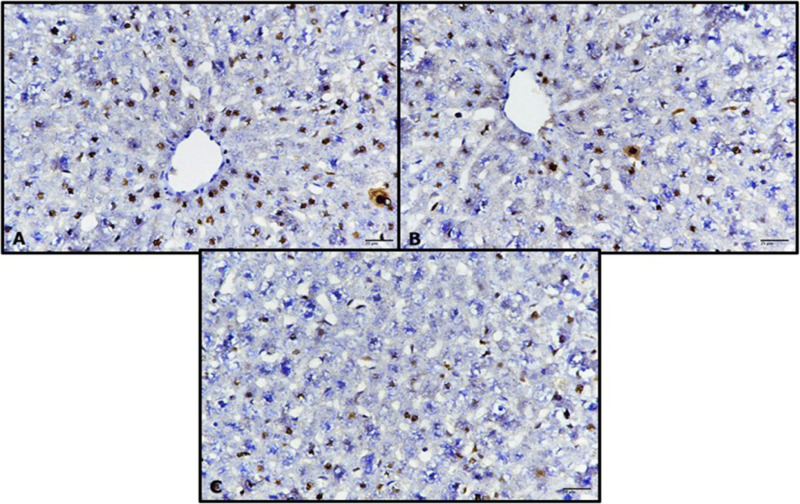
Photomicrographs of rat livers stained immunohistochemically against p53 showing weak immune response. (A) untreated control rats, (B) rats treated with only raspberry ketone (RK) for 1 h, and (C) rats treated with only RK for 24 h (ABC, 400×).

**Fig 12:**
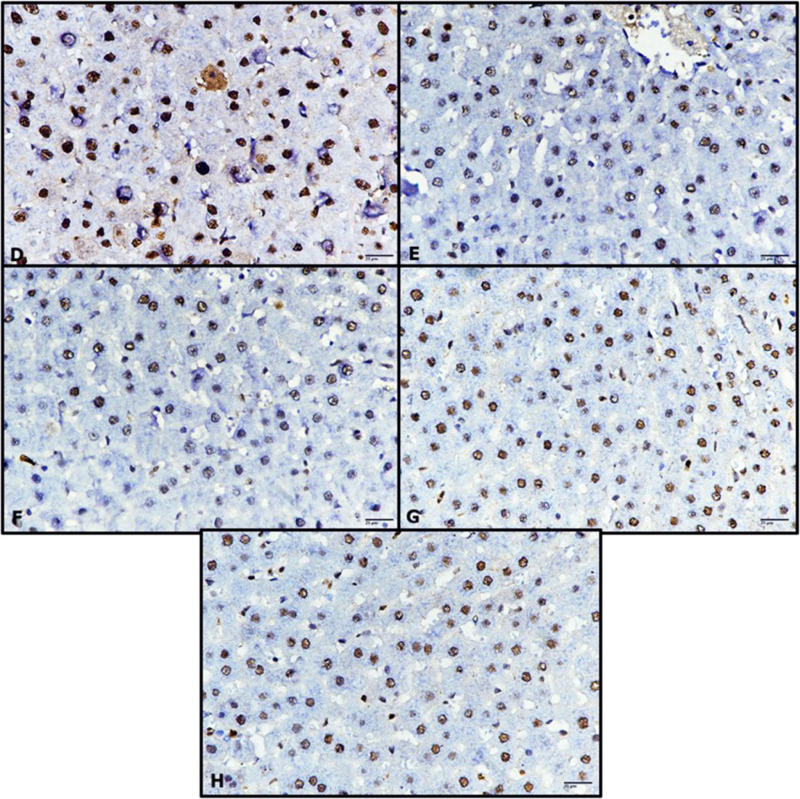
Photomicrographs of rat livers stained immunohistochemically against p53. (D) untreated diabetic rats showing strong immune response, (E) diabetic rats treated with raspberry ketone (RK) for 1 h, (F) diabetic rats treated with RK for 24 h, (G) diabetic rats treated with metformin for 1 h, and (H) diabetic rats treated with metformin for 24 h (D-H, 400×).

The liver sections from untreated control rats stained immunohistochemically against p53 exhibited a weak immune response (Fig 11A). Similarly, the livers of rats treated with only RK for 1 h and 24 h showed a weak immune reaction similar to the control group (Figs 11B and C). In contrast, the livers of the untreated diabetic rats exhibited strong immune reactivity against p53 (Fig 11D), whereas those treated with RK or metformin for 1 h and 24 h showed a reduced incidence of p53 (Figs 12E–H), which indicated its hepatoprotective effects.

## Discussion

We analyzed the effects of RK on streptozotocin-induced diabetic male rats through various tests and examinations. These included assessments of body weight, blood glucose levels, liver function (ALT and AST), lipid profiles (total cholesterol, triglyceride, LDL, and HDL), as well as histopathological and immunohistochemical examinations. Additionally, oxidative stress markers (lipid peroxidation, NOx, and glutathione) and antioxidant defense enzyme activities (catalase and SOD) were evaluated. Furthermore, DNA damage analysis was performed to explore the potential protective role of RK.

In streptozotocin-induced diabetic rats, we observed a significant increase in blood glucose, ALT, AST, triglyceride, and LDL levels and high activity of lipid peroxidation, NOx, catalase, and SOD compared to the control group. However, there was a significant decrease in the activity of glutathione. Notably, no significant changes were noted in the level of total cholesterol. Our immunohistochemical examination revealed that the untreated diabetic group (albino rats) expressed high *p53* levels in the liver in both time intervals, compared to the control group, indicating cellular stress and damage in the diabetic liver. DM is characterized by a lack of insulin, leading to high blood glucose levels, known as hyperglycemia [1,40]. In this study, both streptozotocin-treated and untreated diabetic groups exhibited significantly increased blood glucose levels, confirming the diabetic condition. However, streptozotocin-induced diabetic rats treated with RK or metformin showed a significant decrease in the blood glucose level. Traditional plants and herbal medicines exhibit antidiabetic activities. However, the World Health Organization has recommended further evaluation of these treatments for diabetes [41,42]. The focus on traditional plants aims to identify plant products that are less toxic than currently used drugs [2,11].

The common red raspberry, a fruit of the genus *R. idaeus*, possesses both nutritional and medicinal characteristics. It is rich in micronutrients, dietary fibers, and phenolic components. RK, a compound found in red raspberries, contains phytochemicals, which offer various health benefits. These phytochemicals assist in reducing the risk of chronic diseases, such as cardiovascular diseases, obesity, cancer, and DM [43].

The ALT and AST levels are crucial biomarkers of liver health, with elevated levels indicating liver injury [44]. In this study, the streptozotocin-treated group showed significantly increased ALT and AST levels, indicative of liver damage. However, RK treatment markedly reversed these effects, reducing the levels of liver enzymes (ALT and AST), whereas diabetic rats treated with only metformin exhibited normal levels. These findings suggest that RK plays a protective role against liver injury caused by streptozotocin-induced diabetes in rats, as evidenced by its ability to mitigate the morphological and biochemical changes associated with this disease. Cardiovascular risk assessment often includes measuring serum lipid profiles, which encompass total cholesterol, HDL, LDL, and triglyceride levels [45]. The streptozotocin-induced diabetic rats showed significantly higher TG, HDL, and LDL levels than the controls. However, treatment with RK or metformin significantly reduced these serum lipid levels compared to the diabetic group. These results suggest that RK effectively modulates lipid levels in the blood. This aligns with the findings of Wang *et al*. and Ma *et al*. [46,47]. Notably, RK treatment did not significantly affect total serum cholesterol levels in this study.

Immunohistochemical examination of P53 in the livers of untreated diabetic rats revealed strong immune reactivity against P53, whereas treatment with RK or metformin for 1 and 24 h showed a reduced incidence of P53. These results are consistent with the findings of Fouad *et al*. [26], who investigated the hepatoprotective activity of RK against acute liver damage caused by CCl4. RK exhibited antiapoptotic activity, improved hepatic oxidative stress, and inhibited inflammation and apoptosis [26]. Additionally, Wang *et al*. [46] reported that RK reduced serum TNF-α content, indicating its ability to decrease TNF-α release. These results showed the hepatoprotective effects of RK against streptozotocin-induced type 2 diabetes in male rats.

Free radicals and other non-radical reactive derivatives, collectively known as ROS and reactive nitrogen species, are produced during normal cellular metabolism. These molecules, characterized by high reactivity, can react with lipids, proteins, and DNA, leading to various biological effects. While moderate levels of free radicals play a role in fighting against infectious agents, high levels can cause damage through oxidative stress. There is a direct relationship between diabetes and oxidative stress. Oxidative stress biomarkers, which indicate the presence of ROS, were measured in diabetic rodents [48]. The most common ROS that damage cellular components include hydrogen peroxide (H_2_O_2_), hydroxyl radical (•OH), and superoxide radical (O_2_•) (lipoperoxidation) [49]. Oxidative stress contributes to the progression of aging, diseases such as cancer, atherosclerosis, and diabetes, and cell damage and death through excessive ROS formation [50].

To counteract the overproduction of ROS and oxidative stress, cells use various defense mechanisms, including antioxidant enzymes. SOD and catalase are part of the natural antioxidant defense system, crucial for maintaining the intracellular redox equilibrium [51]. SOD catalyzes the dismutation of superoxide radicals into hydrogen peroxide and oxygen, thus preventing the accumulation of hydrogen peroxide [52]. Hyperglycemia disrupts the antioxidant defense system, leading to altered activities of these enzymes, as observed in diabetic animal models with significantly reduced SOD activity [48]. In our study, SOD played a pivotal role in protecting cells against oxidative stress, evident from the increased SOD levels in streptozotocin-induced diabetic rats. However, treatment with RK reduced SOD levels in the diabetic rats, particularly in those treated for 1 and 24 h, with the 1-h treatment being more effective. This is in line with previous study findings [47], which demonstrated RK’s antioxidant properties in protecting the kidneys of high-fat and high-sugar/streptozotocin-induced diabetic rats. In contrast, catalase facilitates the conversion of hydrogen peroxide into oxygen and water, preventing the accumulation of hydrogen peroxide in the body [53]. In our study, streptozotocin-induced diabetic rats exhibited significantly increased catalase levels compared to the control rats, possibly as the body’s initial defense against diabetes-induced oxidative stress. The decrease in catalase activity observed in the RK-treated rats signifies the strong antioxidant action of RK. These results are consistent with those of Khan *et al*. [54], who showed RK’s beneficial effect in decreasing oxidative and inflammatory reactions and preserving the lipid profile, indicating its potential as an effective treatment for myocardial infarction and hepatoprotective effects. However, glutathione peroxidase plays a crucial role in scavenging hydrogen peroxide and lipid peroxides. Lipid peroxidation is an autocatalytic process typically resulting from cell death, leading to peroxidative tissue damage associated with inflammation, aging, and cancer. Malondialdehyde, an end product of this process, serves as an indicator of lipid peroxidation. It is produced from free oxygen radicals generated during oxidative denaturation [55].

Glutathione converts hydrogen peroxide into water and eliminates peroxynitrite and hydroxyl radicals. Reduced GSH is more effective as an antioxidant than total GSH. Additionally, GSH significantly contributes to the cellular clearance of hydrogen peroxide and other hydroperoxides [56]. In our study, hepatic malondialdehyde levels, an indicator of lipid peroxidation, were significantly higher in the diabetic group than in the control group, and reduced glutathione levels were lower in the diabetic group than in the control group. However, the RK-treated groups showed decreased MDA levels and elevated GSH concentration, indicating a protective role against oxidative damage. These results are in line with those of Mehanna *et al*. [57], who demonstrated RK’s ability to decrease MDA levels and increase GSH levels, suggesting RK’s protective role against oxidative damage.

NOx is a highly reactive free radical that acts as a critical signaling molecule and an antioxidant in the body [58]. It plays a role in various physiological functions, such as neural communication, blood pressure regulation, and immune response [59]. However, excessive NOx levels can lead to tissue damage [60]. High NOx levels have been associated with apoptotic cell death and neurodegeneration induced by oxidative stress [61]. The NOx levels were significantly higher in the diabetic group than in the control group. The group treated with only RK showed significantly reduced NOx levels compared to the diabetic group. The metformin-treated group exhibited levels similar to the control group. Additionally, the group treated with only RK showed significantly reduced NOx levels compared to the control group, suggesting RK’s protective effect against oxidative stress. These findings are consistent with those of Mohamed *et al.* [27], who demonstrated that RK reduced the expression of inducible nitric oxide synthase and attenuated cyclophosphamide-induced pulmonary toxicity. Overall, the results indicated that RK treatment significantly affected the activities of various oxidative stress markers compared to the diabetic group, highlighting the protective effects of RK against oxidative stress.

LDH is a crucial enzyme involved in the anaerobic metabolic pathway, becoming active when cells switch to anaerobic respiration in the absence of oxygen. Elevated LDH levels in the blood serve as indicators of various disorders, including liver diseases, heart attack, cancer, and infections such as HIV. LDH activity can be assessed in different samples, such as serum, plasma, tissue, and cells, with assays measuring LDH leakage from damaged tissue. Another useful test is the LDH isoenzymes test, which helps detect the type, location, and severity of tissue damage, indicating multiple causes of tissue damage simultaneously. LDH serves as an indicator of various tissue injuries due to its isoenzyme form, as cells release LDH into the bloodstream when the tissue is damaged, leading to increased serum LDH levels due to cytoplasm loss [62]. In the present study, the serum LDH levels were significantly higher in the diabetic group than in the control group, consistent with the findings of Abbas [63–65], who observed increased LDH activity in streptozotocin-induced diabetic rats compared to normal controls. However, treatment with RK for both 1 and 24 h resulted in significantly decreased LDH levels compared to the diabetic group, while the groups treated with only metformin exhibited LDH levels similar to the control group. These findings suggest that apoptosis contributes to liver injury from diabetes, as indicated by increased DNA fragmentation in streptozotocin-induced diabetic rats, a phenomenon mitigated by RK treatment. RK exhibits various promising biological activities that warrant further exploration for its potential as a future therapeutic agent. Its administration in diabetic disease could ameliorate adverse effects on normal cells and the body. However, further clinical research is necessary to assess the therapeutic efficacy of RK in humans, alongside comprehensive analyses of its toxicity and safety. This study demonstrates the hepatoprotective effect of RK against streptozotocin-induced T2D.

## Conclusions

In our study, streptozotocin-induced diabetes in rats resulted in significant alterations in serum biochemical parameters, lipid profile, oxidative stress markers, immunotoxicity biomarkers, and DNA damage. RK was found to be safe and effective in mitigating streptozotocin-induced hepatic damage. The study results suggest that RK has hepatoprotective effects by reducing the pathological and biochemical changes associated with diabetes, primarily due to its antioxidant and anti-inflammatory properties. Therefore, incorporating RK into the diet of diabetic patients can help prevent complications (hepatocyte damage) associated with diabetes. Additionally, healthy adults may benefit from consuming RK in terms of reduction in the risk of various diseases that can result from the consumption of unhealthy foods.

## Supporting information

S1 File Whole gel pic. (JPG)
