# Supplementary figures and images for: Hepatoprotective activity of raspberry ketone against streptozotocin-induced type 2 diabetes in male rats
Source: PLoS One. 2025 Jun 9;20(6):e0324940. doi: 10.1371/journal.pone.0324940 (PMC12148097; doi:10.1371/journal.pone.0324940)

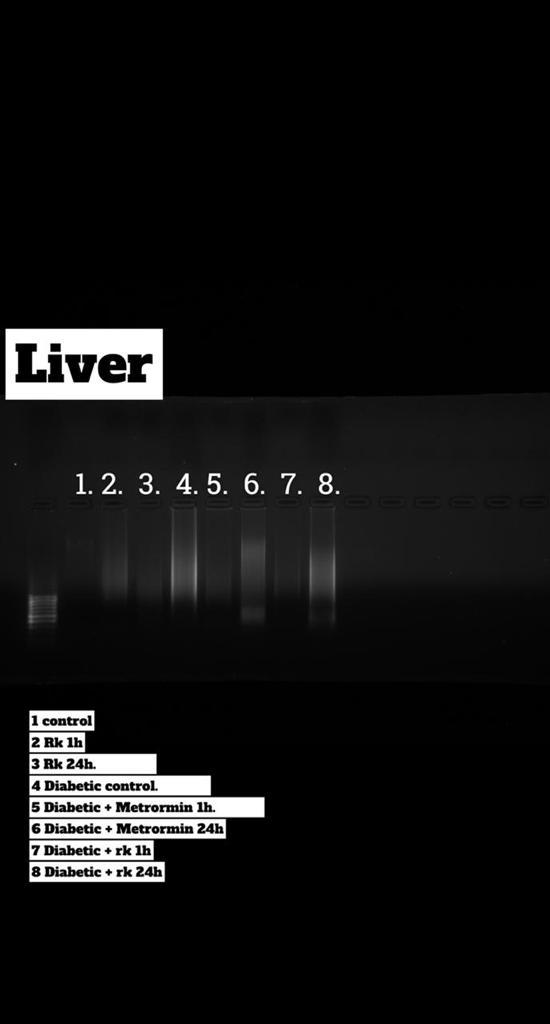

Supplement: S1 File — (JPG) [file pone.0324940.s001.jpg]
